# Vestibulo‐cortical hemispheric dominance: The link between anxiety and the vestibular system?

**DOI:** 10.1111/ejn.13948

**Published:** 2018-06-08

**Authors:** Nadja F. Bednarczuk, Marta Casanovas Ortega, Anne‐Sophie Fluri, Qadeer Arshad

**Affiliations:** ^1^ Division of Brain Sciences Academic Department of Neuro‐Otology Department of Medicine Imperial College London London UK

**Keywords:** anxiety, hemispheric dominance, Spielberger Trait Anxiety Inventory, vestibular cortex

## Abstract

Vestibular processing and anxiety networks are functionally intertwined, as demonstrated by reports of reciprocal influences upon each other. Yet whether there is an underlying link between these two systems remains unknown. Previous findings have highlighted the involvement of hemispheric lateralisation in processing of both anxiety and vestibular signals. Accordingly, we explored the interaction between vestibular cortical processing and anxiety by assessing the relationship between anxiety levels and the degree of hemispheric lateralisation of vestibulo‐cortical processing in 64 right‐handed, healthy individuals. Vestibulo‐cortical hemispheric lateralisation was determined by gaging the degree of caloric‐induced nystagmus suppression following modulation of cortical excitability using trans‐cranial direct current stimulation targeted over the posterior parietal cortex, an area implicated in the processing of vestibular signals. The degree of nystagmus suppression yields an objective biomarker, allowing the quantification of the degree of right vestibulo‐cortical hemisphere dominance. Anxiety levels were quantified using the Trait component of the Spielberger State‐Trait Anxiety Questionnaire. Our findings demonstrate that the degree of an individual’s vestibulo‐cortical hemispheric dominance correlates with their anxiety levels. That is, those individuals with greater right hemispheric vestibulo‐cortical dominance exhibited lower levels of anxiety. By extension, our results support the notion that hemispheric lateralisation determines an individual’s emotional processing, thereby linking cortical circuits involved in processing anxiety and vestibular signals, respectively.

## 1 INTRODUCTION

A considerable degree of overlap between anxiety and dizziness is seen in clinical practice, which thereby confounds patient diagnosis in both psychiatric and neuro‐otology clinics (Staab, 2014; Staab & Ruckenstein, 2010; Thong, Wood, Lo, & Knight, 2007). The link between anxiety and the vestibular system that are both vital processes in balance control was initially described in the 19th century (Gowers, 1887). Since then, a close interaction of these two systems has been highlighted (Balaban, Jacob, & Furman, 2011; Riccelli et al., 2017; Staab, Balaban, & Furman, 2013; Viaud‐Delmon, Venault, & Chapouthier, 2011). Examples of such reciprocal influences include previous findings which demonstrate that patients suffering from vestibular dysfunction are at higher risk of developing anxiety disorders compared to healthy individuals(Best, Eckhardt‐Henn, Tschan, & Dieterich, 2009), and that following an acute unilateral vestibular loss, outcome is most aptly predicted by the patients anxiety level and bodily vigilance status(Cousins et al., 2013). Furthermore, patients with anxiety disorders also frequently complain of greater sensitivity to vestibular stimuli (Staab, Rohe, Eggers, & Shepard, 2014).

Previous findings have separately revealed the functional importance of hemispheric lateralisation for cortical processing of both vestibular and anxiety signals (Balaban, 2002; Carmona, Holland, & Harrison, 2009). With respect to anxiety, the valence theory of emotional processing stipulates that the right hemisphere is specialized for the processing of negative emotions, whereas the left hemisphere predominantly processes positive emotions (Ehrlichman, 1987; Silberman & Weingartner, 1986). Similarly, lateralisation of cortical processing has also been demonstrated in the vestibular system, and functionally, we have recently illustrated the role of such right hemispheric vestibulo‐cortical dominance upon the modulation of vestibular‐guided behaviour, for both brainstem (Vestibulo‐ocular reflex) and cortically mediated (vestibulo‐perceptual) vestibular thresholds.

Based upon the above findings, we propose that hemispheric lateralisation acts as a possible link between anxiety networks and the vestibular system. Specifically, we postulate that an individual’s level of nonsituational (trait) anxiety may be linked to the extent of lateralisation of the vestibular cortex in terms of the degree of right hemisphere vestibulo‐cortical dominance. To test this prediction, we assessed both nonsituational anxiety levels and the degree of right hemispheric vestibulo‐cortical dominance in healthy individuals. Anxiety levels were determined using the trait component of the Spielberger State‐Trait Anxiety inventory (Spielberger, Vagg, Barker, Donham, & Westberry, 1980). Hemispheric lateralisation of the vestibular system was determined using a biomarker that assesses the degree to which a caloric‐induced vestibular nystagmus is suppressed following modulation of cortical excitability using trans‐cranial direct current stimulation (tDCS) applied over the posterior parietal cortex(Arshad et al., 2015), which is a key area in the vestibular cortical network. In conjunction, these measures will allow us to gage the nature of the relationship between anxiety networks and the vestibular system.

## 2 METHODS

### 2.1 Participants

Sixty‐four healthy, right‐handed individuals were recruited (mean age: 22.5, range: 20–33, 32 male). Thirty‐two subjects were allocated to the experimental group (cathodal stimulation), with the remaining 32 subjects forming the control group (anodal stimulation). Subjects were excluded on a basis of any electric stimulation contraindications such as a personal or family history of epilepsy or any metal implants. Furthermore, subjects were excluded if there was any past or existing psychiatric, otological, ophthalmological or neurological disorder. Practically this was performed during a screening session before participants were recruited into the study by asking them directly, if they were currently or ever had been in the past, under the care of a specialist doctor for any eye, ear, brain or psychiatric condition. None of the participants were taking psychoactive medication. All subjects provided written informed consent as approved by the local ethics committee.

### 2.2 Anxiety measurement

Anxiety was assessed using the Trait component of the Spielberger Anxiety Questionnaire (Spielberger, 2010; Spielberger et al., 1980). All Subjects were granted privacy during completion of the questionnaire. Participants were informed that they would receive a unique key code to ensure participant anonymity and confidentiality.

The Trait component questionnaire contains 20 statements ascertaining various aspects of an individual’s propensity to anxious traits (Julian, 2011). Subjects were asked to rate the 20 questions on a scale of 1–4 (with 1 = never, 2 = sometimes, 3 = very often, 4 = always). Questions are aimed at assessing feelings of stress, worry and other manifestations of anxiety on a day‐to‐day basis, for example, “I feel like a failure,” “I am happy” or “I worry too much about things that really doesn’t matter” (Spielberger & Sydeman, 1994). Accordingly, the questionnaire yields a maximum score of 80 and a minimum of 20, with higher scores representing greater levels of anxiety. As the Trait component assesses stable rather than situational anxiety levels, the experimental protocol implemented (see below) should not impact anxiety scores. However, to explicitly eliminate any potential carry‐over effects of the experimental protocol (i.e., either brain stimulation or vestibular stimulation), half the participants in both the anodal and cathodal groups completed the questionnaire before and the other half after the experiment. Note, we observed no significant difference between these two groups when comparing the scores obtained either before or after vestibular and brain stimulation respectively (*p* > 0.05, unpaired *t* test).

### 2.3 Trans‐cranial Direct Current Stimulation (tDCS)

Trans‐cranial Direct Current Stimulation was applied using a battery‐driven stimulator (neuroConn GmbH, ilmenau, Germany) for 15 min with a current of 1.5 mA (ramp‐up and fade‐out time of 10 s). Stimulation was applied over the left posterior parietal cortex (P3; 10–20 international EEG System) with the reference electrode over the ipsilateral deltoid muscle in both the experimental and control condition. This cortical area and montage were selected as it is strongly implicated in vestibular processing and has been shown to modulate vestibular responses (Bednarczuk, Casanovas Ortega, Fluri, Bronstein, & Arshad, 2017). We applied either unipolar cathodal (test condition) or anodal stimulation (control condition), which previous research has shown not to have any effect on vestibular processing and additionally provides an active control for any nonspecific effects associated with electrical stimulation(Arshad, Nigmatullina, & Bronstein, 2013; Arshad et al., 2014, 2015).

### 2.4 Quantification of hemispheric dominance

Caloric irrigations were performed before and after the application of tDCS. Subjects lay supine on a reclined barber’s chair with their head tilted up 30^°^ to obtain maximal horizontal semicircular canal activation. Irrigations were performed separately for both right and left ears, implementing a 5‐min rest period between irrigations to avoid any carry‐over effects, with cold water (30°C) for 40 s with a flow rate of 500 ml/min. We simultaneously recorded the resultant eye movements for 2 min using a head‐mounted, infra‐red Video‐oculography system. Eye movements were analysed automatically by extracting the slow phase components of the vestibular nystagmus which were plotted over time to identify the peak slow phase velocity (SPV). Furthermore, to determine hemispheric dominance, we calculated the average change in the peak SPV following left hemisphere cathodal tDCS (test condition), to provide the nystagmus suppression index, which acted as a surrogate measure of hemispheric dominance (Arshad et al., 2015). Specifically, the formula implemented to calculate the nystagmus suppression index, involved dividing the peak post‐tDCS peak SPV minus the peak pre‐tDCS SPV by the peak pre‐tDCS SPV × 100. Note, suppression was only expected following cathodal stimulation, but not following anodal stimulation. The greater the nystagmus suppression index, the more right hemisphere dominant that individual is (Arshad et al., 2014, 2016).

To explain the choice of the implemented stimulation paradigm further, it is beneficial to briefly review our previous findings in a sequential order. Application of a bihemispheric montage, namely anodal stimulation of the right hemisphere (posterior parietal cortex; P3/P4) and concurrent cathodal stimulation of the left hemisphere results in suppression of left‐beating vestibular nystagmus (right ear cold irrigations). This nystagmus is predominantly processed by the left hemisphere, but this bihemispheric montage has no effect upon the right beating nystagmus produced by left ear cold water irrigations, which are predominantly processed in the right hemisphere. Reversing the polarities in the bihemispheric montage has no modulatory effect upon the vestibular ocular reflex (VOR). To elucidate the activated electrode in the bihemispheric montage, we applied unipolar tDCS and observed that the modulation was attributable to cathodal stimulation of the left hemisphere (Arshad et al., 2014).

Critically, our previous results demonstrate that right hemisphere cathodal stimulation does not modulate the vestibulo‐ocular reflex in right‐handed individuals, and this lack of modulation is surprising as inhibition of the right posterior parietal cortex disrupts interhemispheric interactions and should theoretically affect vestibular processing (Arshad et al., 2014). However, this lack of modulation can be reconciled with the notions that in right handers, the right hemisphere is able to better compensate than the left for the induced inhibition via cathodal stimulation, attributable to its dominance for vestibular processing (Arshad et al., 2014; Dieterich et al., 2003), and the ongoing functional asymmetry between the two parietal cortices enabling the right hemisphere to exert stronger inhibition over the left hemisphere facilitated by asymmetric parietal interhemispheric connections (Koch et al., 2011).

Accordingly, our previous findings imply that cathodal stimulation over the left hemisphere, not only inhibits function in the left hemisphere but also potentiates right hemisphere dominance, and thus renders the left hemisphere less able to process the vestibular nystagmus. Thus, greater vestibular nystagmus suppression following left parietal cathodal tDCS implies increased right hemispheric vestibulo‐cortical dominance.

## 3 RESULTS

To assess the relationship between an individual’s anxiety and the degree of vestibular hemispheric dominance, we correlated Trait anxiety scores for each individual with their nystagmus suppression index following cathodal tDCS to the left posterior parietal cortex.

**Figure 1:**
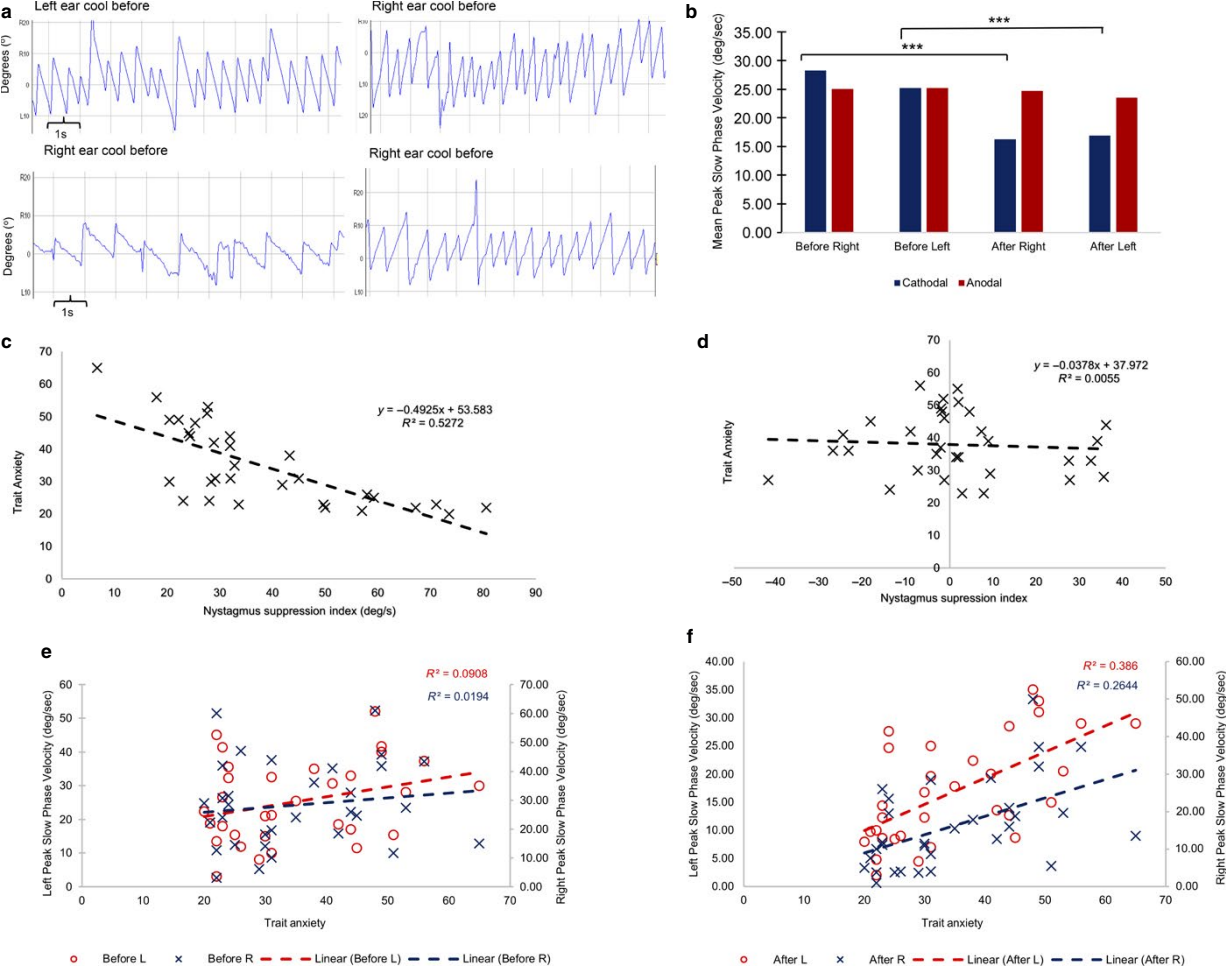
(a) Example raw data recordings of a caloric‐induced nystagmus in one participant before and after the application of cathodal trans‐cranial direct current stimulation (tDCS) for both right and left cold water irrigations. Traces are displayed fir both right and left ear irrigations. The recordings before tDCS are above those recorded after the application of tDCS. The *x*‐axis represents time, with one large square representing 1 s. The *y*‐axis represents the degrees of eye movement either right (R) or left (L) from the centre (0^°^). To measure the slow phase velocity (SPV) of a nystagmus, one utilises the slope of the nystagmus in its slow phase. In these recordings once can clearly see a less steep slope, and therefore a suppressed SPV, of the nystagmus following cathodal tDCS. (b) Mean peak SPV recordings pre‐ and post‐ the application of both cathodal and anodal tDCS. The *x*‐axis represents the different groups of irrigation (i.e., right and left irrigations before and after tDCS) for both cathodal and anodal stimulation. The *y*‐axis represents the mean peak SPV (degrees/s). There is a suppression in the mean peak SPV only for right and left irrigations following cathodal tDCS. Anodal stimulation has no impact upon the mean peak SPV (***p < 0.001). (c) Relationship between the degree of Right Hemisphere Dominance and the Trait Anxiety Scores in the cathodal (experimental) group. The *x*‐axis represents the Nystagmus suppression index (i.e., the objective biomarker for right hemispheric dominance). A greater nystagmus suppression relates to a greater degree of right hemisphere dominance, whilst less nystagmus suppression relates to less right hemisphere dominance. This is correlated with the Trait Anxiety Score, as represented on the *y*‐axis. A negative relationship exists between the two, with a greater extent of right hemisphere dominance relating to lower Anxiety scores. A linear line of best‐fit is drawn with the correlation achieving a Pearson Correlation Coefficient (*R* ^2^) of 0.52715. Total number of subjects = 32. (d) Relationship of the degree of Right Hemisphere Dominance and the Trait Anxiety Score for the anodal (control) group. The *x*‐axis represents the Nystagmus suppression index (i.e., the objective biomarker for right hemispheric dominance). A greater nystagmus suppression relates to a greater degree of right hemisphere dominance, whilst less nystagmus suppression relates to less right hemisphere dominance. No relationship was found to exist between the nystagmus suppression index and the Trait anxiety in the anodal group (*R* ^2^ = 0.0055) Total number of subjects = 32. (e) Relationship of right (blue) and left (red) ear peak SPV recordings before the application of cathodal tDCS and the corresponding Trait anxiety scores. The *x*‐axis represents the Trait anxiety scores of the participants in the cathodal (experimental) group. The left (primary) *y*‐axis represents the peak SPV for left ear cold water caloric irrigations (degrees/s). The right (secondary) *y*‐axis represents the peak SPV for the right ear cold water caloric irrigations (degrees/s). No relationship exists between participants’ anxiety level and their caloric‐induced nystagmus prior to the application of tDCS (Left: *R* ^2^ = 0.09075, Right: *R* ^2^ = 0.01939). (f) Relationship of right (blue) and left (red) ear peak SPV recordings after the application of cathodal tDCS and the corresponding Trait anxiety scores. The *x*‐axis represents the Trait anxiety scores of the participants in the cathodal (experimental) group. The left (primary) *y*‐axis represents the peak SPV for left ear cold water caloric irrigations (degrees/s). The right (secondary) *y*‐axis represents the peak SPV for the right ear cold water caloric irrigations (degrees/s). No relationship exists between participants’ anxiety level and their caloric‐induced nystagmus prior to the application of tDCS (Left: *R* ^2^ = 0.38605, Right: *R* ^2^ = 0.26436 [Colour figure can be viewed at http://wileyonlinelibrary.com]

First, it is important to confirm a significant impact of tDCS upon vestibular function, specifically assessed by the suppression of the mean peak SPV of a caloric‐induced nystagmus, as illustrated by the raw vestibular nystagmus traces from one of our participant’s (Figure 1a). Pre‐tDCS, the mean peak SPV for the right cold irrigations was 28.28°/s in the cathodal group and, 25.03°/s in the anodal group. For left cold irrigations the mean peak SPV was 25.23°/s in the cathodal group and 25.24°/s in the anodal group pre‐tDCS. Post‐tDCS, the peak mean SPV for right cold irrigations was 16.25°/s in the cathodal group and 24.70°/s for the anodal group. For left cold irrigations post‐tDCS, the mean peak SPV was 16.91°/s in the cathodal group and 25.53°/s for the anodal group. These recordings now allowed for the calculation of the nystagmus suppression index. We only observed a significant suppression of vestibular nystagmus following cathodal stimulation of the left posterior parietal cortex for both right (*p* = 6.07 × 10^−7^, paired *t* test) and left irrigations (*p* = 5.35 × 10^−7^, paired *t* test), as shown in Figure 1b. In contrast, anodal stimulation did not induce a significant vestibular nystagmus suppression for neither left (*p* = 0.79, paired *t* test) nor right (*p* = 0.13, paired *t* test) irrigations.

We observed a significant positive correlation (Pearson’s correlation coefficient (*R* ^2^) = 0.525, *p* = 0.000003; Figure 1c) in the experimental group (i.e., cathodal stimulation). That is, more right hemisphere dominant participants (i.e., greater nystagmus suppression) scored lower on the Trait Anxiety Questionnaire. Less right hemisphere dominant individuals (i.e., lower nystagmus suppression) scored more highly on the questionnaire. As expected, no correlation was observed in the control group [i.e., anodal stimulation] (Pearson’s correlation coefficient *R* ^2^ = 0.005, *p* = 0.687; Figure 1d). Furthermore, supporting the relationship we report is the fact that we observed no significant correlation between trait anxiety scores and pre‐cathodal tDCS peak mean SPV for either right cold (Figure 1e, *R* ^2^ = 0.01939, *p* = 0.447) or left cold irrigations (Figure 1e, *R* ^2^ = 0.09075, *p* = 0.094). Post‐tDCS a significant correlation can be seen between trait anxiety scores and the peak mean SPV for both right cold (Figure 1f, *R* ^2^ = 0.26436, *p* = 0.002610) and left cold irrigations (Figure 1f, *R* ^2^ = 0.38605, *p* = 0.000148). One would expect a weak correlation with exist between trait anxiety and the mean peak SPV following the application of tDCS, as this reflects the cortical influences of tDCS. This correlation is strengthened by the full calculation of the nystagmus suppression index by incorporating the pre‐tDCS SPV values.

It is important to recall that in this study, we only tested normal subjects. Considering a suggested cut‐off point of 39–40 to detect clinical manifestations of anxiety in the Trait Spielberger Questionnaire (Julian, 2011; Knight, Waal‐Manning, & Spears, 1983), of our 64 participants, 25 (N.B. 12 in the cathodal group and 13 in the anodal group), had a score higher than 40. The fact that these healthy controls have a score higher than the suggested cut‐off, does not imply that there is an undetected anxiety disorder present, but simply that these subjects are more anxious on a day‐to‐day basis but are able to function normally. Critically, there was no significant difference in Trait anxiety scores when comparing between the 32 subjects in the experimental group (cathodal stimulation) and the 32 subjects in the control group (anodal stimulation) ((*p* = 0.276, paired *t* test).

Furthermore, given that the literature has suggested gender‐related differences in emotional processing (Wager, Phan, Liberzon, & Taylor, 2003), we sought to investigate any such differences in our data set. We observed no significant gender‐related differences in anxiety or hemispheric dominance (*p* > 0.05, paired *t* test).

## 4 DISCUSSION

Our results demonstrate that an individual’s degree of vestibulo‐cortical hemispheric dominance correlates with their anxiety levels. Specifically, we observed that greater right hemisphere dominance was associated with lower anxiety, whereas increased anxiety was found in less right hemisphere dominant individuals. Our results highlight the interplay between vestibular processing and anxiety networks (Riccelli et al., 2017), but also implicate the importance of hemispheric lateralisation upon influencing these systems.

The role of hemispheric lateralisation in emotional processing has been repeatedly demonstrated in the literature. Lesion studies have shown that left hemisphere damage is associated primarily with depressive symptoms (Bolla‐Wilson, Robinson, Starkstein, Boston, & Price, 1989; Starkstein et al., 1989). Contrastingly, individuals with right hemisphere lesions reported difficulties in emotional facial recognition tasks(Bourne & Watling, 2015; Mandal, Mohanty, Pandey, & Mohanty, 1996). Furthermore, the right hemisphere has been implicated both in the major processing and displaying of all affective states as demonstrated by more intense facial expression of emotion on the left side of the face (Alves, Fukusima, & Aznar‐Casanova, 2008; Killgore & Yurgelun‐Todd, 2007; Sackeim, Gur, & Saucy, 1978). More specifically, patients with brain tumours in the right hemisphere score more highly on anxiety scales than patients with left hemisphere tumours. Notably, following tumour removal the elevated anxiety scores return to baseline values (Mainio et al., 2003). These findings are supported by the valence theory of emotional processing, which proposes the predominant processing of negative emotions, such as fear, in the right hemisphere, whilst positive emotions, such as happiness, are processed in the left hemisphere (Davidson, 1995). Thus, it appears to be the case that disruption of normal hemispheric interactions results in maladaptive emotional processing functionally manifesting as anxiety (Davidson, Fedio, Smith, Aureille, & Martin, 1992).

Hemispheric interactions have been suggested to underpin not only emotional processing (Davidson, 1995), but also vestibulo‐cortical control (Arshad, 2017; Bednarczuk et al., 2017). With respect to vestibular processing, it has been shown that in right‐handed individuals predominant processing of vestibular signals occurs in the right hemisphere, whereas in left‐handers, the left hemisphere is dominant (Arshad et al., 2013; Dieterich et al., 2003; Nigmatullina et al., 2016). Furthermore, the degree of right hemisphere dominance in the vestibular system has been shown to influence both cortical (i.e., early visual cortex excitability) and brainstem (i.e., modulation of the vestibulo‐ocular reflex) functions (Arshad et al., 2015). Additionally, right hemisphere vestibulo‐cortical dominance has been shown to influence the modulation of vestibular thresholds following a change in cortical excitability (Bednarczuk et al., 2017). Finally, vestibulo‐cortical hemispheric lateralisation not only influences vestibular function, but it has also been shown to influence cognitive processes such as numerical processing and economic decision‐making (Arshad et al., 2016). Accordingly, vestibulo‐cortical hemispheric dominance appears to have influences upon brain functions far beyond the conventional function of the vestibular system as demonstrated by previous and current findings.

An outstanding question is why an association exists between a low‐level brainstem‐mediated VOR and anxiety, an affective measure. To address this, it is firstly important to note that the baseline measures of the VOR do not correlate with anxiety scores, as shown in Figure 1e. Rather, it is the degree of cortically mediated suppression of the brainstem VOR that correlates with the anxiety scores, implying a cortically‐controlled re‐scaling of brainstem‐cortical interactions. In line with this, our previous findings have demonstrated that the nystagmus suppression index correlates with cognitive biases such as numerical magnitude perception and spatial attention (Arshad et al., 2015, 2016), further supporting the notion of a brainstem‐cortical re‐scaling network.

The above‐reviewed findings regarding hemispheric influences upon anxiety and vestibular processing are in concordance with our present findings. That is, we observed that individuals with greater right hemispheric vestibulo‐cortical dominance are less anxious compared to less right hemisphere dominant individuals. Based upon the neuro‐anatomical and functional overlap between anxiety and vestibular systems, we suggest a possible role of hemispheric dominance in mediating the extent of anxious feelings and the perception of dizziness. Further support for this link is provided by clinical reports that illustrate an overlap between anxiety and dizziness (Balaban et al., 2011; Staab, 2014) such as the finding that in chronic subjective dizziness following vestibular dysfunction is linked to elevated anxiety levels (Staab, 2014; Staab & Ruckenstein, 2005).

A factor not explicitly considered in our present study is handedness, which is known to influence cerebral hemispheric dominance. As aforementioned, hemispheric lateralisation influences not only anxiety (i.e., in right handers, the right hemisphere has been shown to mainly handle negative affective emotions) (Silberman & Weingartner, 1986), but also cortical processing of vestibular signals (Arshad et al., 2013, 2015, 2016; Bednarczuk et al., 2017). Although it is important to consider that handedness can impact the asymmetry present in cerebral processing, it does so to varying extents (Costanzo et al., 2015). Our study considered only right‐handed subjects whom demonstrated varying degrees of right hemisphere dominance throughout. Therefore, implementing the subject‐specific nystagmus suppression index as an objective biomarker for each individual’s hemispheric dominance can provide a controlled quantifiable measure of its respective contribution to anxiety.

In conclusion, our findings demonstrate that vestibulo‐cortical hemispheric dominance correlates with trait anxiety scores. Future research should aim to investigate the influence of hemispheric lateralisation upon associated anxiety in dizzy patients.

## CONFLICT OF INTEREST

The authors declare no competing financial interests.

## DATA ACCESSIBILITY

Data are available from the corresponding author on request.

## AUTHOR CONTRIBUTIONS

N.F.B. conceptualised study, data collection, data analysis, wrote manuscript, prepared figures; M.C.O. data collection, data analysis, revised manuscript; A.S.F. data collection, data analysis, revised manuscript; Q.A. conceptualised study, wrote manuscript and supervised study.
