# Supplementary material for: Vestibulo‐cortical hemispheric dominance: The link between anxiety and the vestibular system?
Source: Eur J Neurosci. 2018 Jun 8;47(12):1517–24. doi: 10.1111/ejn.13948 (PMC6099323; doi:10.1111/ejn.13948)
Supplement: Supplementary file 1 [file EJN-47-1517-s001.pdf]

EJN European Journal of Neuroscience FENS Federation of European Neuroscience Societies

Vestibulo-cortical Hemispheric Dominance: the link between Anxiety and the Vestibular System?

Nadja F. Bednarczuk, Marta Casanovas Ortega, Anne-Sophie Fluri and Qadeer Arshad1a

Review timeline:	Submission date:	07 August 2017
	Editorial Decision:	03 October 2017
	Revision Received:	09 January 2018
	Editorial Decision:	26 February 2018
	Revision Received:	09 March 2018
	Accepted:	04 April 2018

Editor: Christoph M. Michel

1st Editorial Decision 03 October 2017

Dear Dr. Arshad,

Your manuscript was reviewed by two external reviewers as well as by the Section Editor, Prof. Christoph M. Michel, and ourselves. Based on these reviews, we regret to inform you that we are not able to accept your manuscript for publication in EJN in its present form. However, the research described in your manuscript is potentially of interest and therefore we invite you to resubmit a much revised version. As you will read, both reviewers expressed major concerns regarding the manuscript. The concerns are related to the unilateral stimulation, the application of the questionnaire after stimulation, the enhanced anxiety levels of a large number of subjects, and the missing description of the regression analysis. These and other points raised by the reviewers need to be carefully addressed in a resubmission.

Please also attend to the following issues:
1) Provide a clear data sharing statement, and we strongly encourage that you place your data on a public access repository (e.g. Figshare)
2) Author contributions need to be explicitly included in the manuscript itself
3) Provide the full name of the ethics committee that approved the procedures of the study
4) Do not report P-values as inequalities (see EJN guidelines)

Obviously, a resubmission of your manuscript could only be considered on the basis of extensive revisions, requiring the description of new evidence and/or additional analyses of your data. When you prepare your re-submission, please consult the Author's Checklist provided at the bottom on this email, notably with regard to characterization of reagents and reporting of statistical analyzes in the Results section and Figure legends.

Thank you for submitting your work to EJN.

Kind regards,

John Foxe & Paul Bolam
co-Editors in Chief, EJN

Reviews:

Reviewer: 1 Alexander Tarnutzer (University Hospital Zurich, Switzerland)

Comments to the Author
Reviewer's comments EJN

In this study, Bednarczuk and colleagues report on hemispheric dominance of vestibular processing and its role for anxiety levels. Specifically, the authors explored the interaction between cortical vestibular processing and anxiety by correlating anxiety levels with the degree of hemispheric lateralization in healthy right-handed subjects. TDCs was used to modulate cortical excitability and cold-water caloric irrigation was used to determine the extent of hemispheric lateralization afterwards. The Trait component of the Spielberger State-Trait anxiety questionnaire was applied to estimate anxiety levels. In the experimental group (with cathodal TDCs) those individuals with greater right-hemispheric dominance demostrated lower levels of anxiety. The authors conclude that their findings support the notion that hemispheric lateralization plays an important role in the individual's emotional processing.

The link between anxiety/depression and chronic dizziness is clinically important and so far only partially understood. The study therefore addresses an interesting and also clinically relevant question. However, I do see several significant limitations of the work presented here. This includes both the introduction (hypothesis missing), the methods (no description of regression analyses applied), the results (see below) and their interpretation.

First and probably most important is the fact that more than one fourth of all subjects had anxiety levels that were rated abnormal. Surprisingly, all of these subjects were in the experimental group according to figure 1. This may have resulted in a bias and a false positive correlation between levels of hemispheric dominance and anxiety levels. This is worriesome and needs to be explained by the authors'. Currently, it remains unclear to me whether the authors indeed found evidence for a correlation between hemispheric lateralization and anxiety levels in their study.
Second, I am missing any reporting of raw data and data on caloric irrigation.

Specific comments

Abstract:
None

Introduction:
General comment: I am missing a hypothesis and predictions based on the hypothesis stated.

Methods:
Page 4, third paragraph: „Irrigations were performed in both right and left ears with cold water (30oC) for 40s ..."
This sentence might be misleading. I assume that both right and left ears refers to sequential caloric irrigation, not bilateral caloric irrigation at the same time.

Results:
Page 5, 2nd paragraph: How did the authors evaluate psychiatric disorders in the controls before inclusion to the study?

I am worried about the scores in the Trait Spielberger questionnaire reported. With more than 25% of the healthy controls having a score that indicates anxiety disorder, the questionaire used is either not suitable or the cut-off point selected is too low.

when looking at figure 1, it seems that all 12 controls with scores >40 were in the experimental group. this is worriesome and I am surprised that statistical analysis of anxiety scores were not different amongst the experimental and the control group.

Also I wonder why the authors emphasize the value of 65. Is this another cut-off point?

If the authors do the correlation analysis again with the 10 subjects that had normal scores in the experimental group, is the reported correlation still present? this would help in confirming the correlation reported here in healthy control subjects.

Page 5, 2nd paragraph: „Critically, there was no significant difference in Trait anxiety between the two groups (p>0.05, unpaired t-test)."
is this referring to the 22 patients with anodal vs. the 22 patients with cathodal stimulation?
How was the distribution of scores amongst these two groups?

Discussion:
Page 6, 2nd paragraph from bottom: „...which should be further explored as a potential therapeutic option, especially in patients with chronic dizziness"
this conclusion comes out of the blue. suddenly the authors speak about the treatment of chronic dizziness. Please clarify.

Figure legend Fig. 1:
I am missing a description of the methods used to perform the regression analysis. Was an approach used that takes the fact into account that both variables were dependent, i.e. measured with noise? normal regression analysis does require one independent variable.

Reviewer: 2 Christian Pfeiffer (Ecole Polytechnique Federale de Lausanne, Switzerland)

Comments to the Author
General
This study investigates the relationship between hemispheric dominance in vestibular processing and anxiety trait. Vestibular-caloric nystagmus suppression induced by left anodal transcranial direct current stimulation positively correlated with anxiety questionnaire scores. The results seem to suggest that stronger right-hemispheric specialization relates to lower trait anxiety.
The research question is interesting and novel. However, the manuscript presents with deficiencies in experimental design and over-interpretation of the results. I do not recommend publication of the manuscript, and dataset, in its current version.

Major

1. A major concern is that vestibular stimulation (and/or TDCS) may have affected questionnaire ratings. The authors presented questionnaires either before or after vestibular/TDC stimulation to subjects who received either anodal or cathodal TDCS. Results were analyzed across subjects separately for the anodal and cathodal group. Was there a difference in questionnaire scores between patients receiving the questionnaire before or after vestibular stimulation? .. between those receiving anodal vs. cathodal stimulation when completing the questionnaire after the experiment? Was the proportion of subjects who received the questionnaire before vs. after balanced in the anodal vs. cathodal groups? These possible confounds could have been addressed by administering the questionnaire twice (before and after) the experiment. Post-hoc analysis, giving matched and sufficiently large sample sizes across subgroups should be included.

2. Another concern is that authors exclusively applied TDCS to the left hemisphere, however, results are discussed with respect to (not tested) right hemispheric processing. It may be one of many possible causes underlying the observed effect, however, based on unilateral left stimulation alone this conclusion cannot be drawn. A cross-over design including unilateral left, unilateral right, as well as bilateral stimulation (as used in a previous publication by the same authors, Arshad et al. 2014 Brain Stimulation) would have been desirable in order to provide evidence to the hemispheric specialisation hypothesis (and in order to address concern 1 regarding the direct effects of TDCS on anxiety scores).

3. Results are largely over-interpreted, e.g.
- page 5: “Our results demonstrate that … hemispheric dominance can predict anxiety levels”. No, “prediction” implies temporal precedence, which cannot be inferred from the correlational results presented here.
- page 6: “.. we observed that a more right hemisphere dominant individual is better able to control negative emotions..”. It is not clear if the questionnaire event tested the control of negative emotions. Authors should report the questionnaire questions, and analysis of sub-scores in order to support their claim.
- page 6, “… our findings suggest that hemispheric dominance is the intricate mechanistic link between anxiety networks and the vestibular system.. “. Based on a rather vague discussion of hemispheric dominance, and the aforementioned concerns, it is unclear how the authors arrive at this conclusion - mechanistic, how exactly?

Minor
page 2, “left posterior parietal cortex, an area heavily implicated in the processing of vestibular signals” is somehow at odds with the notion of right-hemispheric dominance (Dieterich et al. 2003 Cerebral Cortex)

page 2, “Our findings demonstrate that … hemispheric dominance can directly predict anxiety levels”: The term “prediction” implies temporal precedence, which is neither tested nor shown by the present study.

page 2: “… hemispheric lateralization determines … emotional processing.”: The results are correlational and cannot be interpreted in terms of causality.

page 3. The Introduction is rather weak, as it remains vague at pointing out the involvement of cortical hemispheres, without clearly showing that the same hemisphere and the same functional networks are involved. Also, the authors should expand the introduction of the background for the left TDCS technique testing hemispheric dominance (e.g. see Arshad et al. 2014 Brain Stimulation).

page 3. “The link between anxiety and vestibular system, …”. It appears the authors refer to two distinct types of systems? Vestibular system, a sensory system processing vestibular afferents, and the anxiety system attributing negative valence to a large variety of sensory inputs?

page 3. “between visuo-vestibular and anxiety system”. Why mention visual processing here? This is not at all tested in the present study, considering the VOR works also in absence of visual inputs.

page 4. Did the authors not administer the “State” component of the questionnaire or missed to report these results? Could they include a listing of all questions, median scores, interquartile range across subjects, and report analysis of sub-scores (e.g. emotion control).

page 4. Authors should also analyse and report peak latency of the slow phase velocity within the 2min recording period between pre- and post-TDCS.

page 4. how was the nystagmus suppression index exactly calculated? by dividing the differences post minus pre by the absolute value of pre, multiplied by 100? Could the authors specify this in the manuscript?

page 5. Could the authors perform additional correlation analysis between the SP peak velocity before TDCS and questionnaire scores, and a separate analysis of SP peak velocity after TDCS and questionnaire scores. No correlation in these analyses would further strengthen support the interpretation that TDCS-induced suppression relates to anxiety scores. However significant correlation with pre-TDCS SPV values could reflect TDCS unrelated effects that may have contributed to the pre-post change index (if used to normalize this difference value).

Figure 1. It seems preferable to show the y-axis on the left-hand side of the plot. The authors may want to include a schematic illustrating the TDCS montages (anodal, cathodal). In the captions, it is not mentioned that only the data from half of the subjects are presented. The same plot should be added for the other (control) group.

EJN European Journal of Neuroscience FENS Federation of European Neuroscience Societies

Authors' Response 09 January 2018

Reviews:
Reviewer: 1; In this study, Bednarczuk and colleagues report on hemispheric dominance of vestibular processing and its role for anxiety levels. The link between anxiety/depression and chronic dizziness is clinically important and so far only partially understood. The study therefore addresses an interesting and also clinically relevant question. However, I do see several significant limitations of the work presented here. This includes the introduction (hypothesis missing), the methods (no description of regression analyses applied), the results (see below) and their interpretation.
We thank the reviewer for his/her overall positive assessment of our manuscript. Regarding the limitations identified, we have now addressed the concerns raised by the reviewer on a point by point basis as set out below.
Q1. First and probably most important is the fact that more than one fourth of all subjects had anxiety levels that were rated abnormal. Surprisingly, all of these subjects were in the experimental group according to figure 1. This may have resulted in a bias and a false positive correlation between levels of hemispheric dominance and anxiety levels. This is worrisome and needs to be explained by the authors'. When looking at figure 1, it seems that all 12 controls with scores >40 were in the experimental group. This is worrisome and I am surprised that statistical analysis of anxiety scores was not different amongst the experimental and the control group. Currently, it remains unclear to me whether the authors indeed found evidence for a correlation between hemispheric lateralization and anxiety levels in their study.
R1. We acknowledge the reviewers concern and have accordingly increased the sample size from 44 subjects to 64 subjects. This was performed in order to ensure that both the experimental and the control group had an equal number of subjects with an anxiety score greater than 40. This ensured that the two groups were equally matched and to avoid the possibility of a bias and false positive correlation (also see response to reviewer #2, comment Q8). We observed, that increasing the sample size to account for any differences did not affect the results we initially reported, as we still observe a correlation between levels of hemispheric dominance and anxiety levels in the cathodal (i.e. experimental) but not in the anodal (i.e. control) group respectively. Please find the above reported changes incorporated on page 9, Paragraph 1 of the revised manuscript. Alternatively, please see below for the change.
"It is important to recall that in the current study we only tested normal subjects. Considering a suggested cut-off point of 39-40 in order to detect clinical manifestations of anxiety in the Trait Spielberger Questionnaire(Knight et al., 1983; Julian, 2011), of our 64 participants, 25 (N.B. 12 in the cathodal group and 13 in the anodal group), had a score higher than 40. The fact that these healthy controls have a score higher than the suggested cut-off, does not imply that there is an undetected anxiety disorder present, but simply that these subjects are more anxious on a day-to-day basis but are able to function normally. Critically, there was no significant difference in Trait anxiety scores when comparing between the 32 subjects in the experimental group (cathodal stimulation) and the 32 subjects in the control group (anodal stimulation) ((p= 0.276, paired t-test)."

Q2, Second, I am missing any reporting of raw data and data on caloric irrigation.
R2. We apologise for this omission and we have now have now included this in the last paragraph of page 7 and the first paragraph of page 8 of the manuscript, or alternatively, please refer to the inserted paragraph below. Further, we have summarised this data graphically in Figure 1B and we now also show illustrations using raw traces of the vestibular nystagmus suppression following cathodal stimulation (Figure 1A).
"Firstly, it is important to confirm a significant impact of tDCS upon vestibular function, specifically assessed by the suppression of the mean peak SPV of a caloric-induced nystagmus, as illustrated by the raw vestibular nystagmus traces from one of our participant's (Figure 1A). Pre-tDCS, the mean peak SPV for the right cold irrigations was 28.28 o/sec in the cathodal group and, 25.03 o/sec in the anodal group. For left cold irrigations the mean peak SPV was 25.23 o/sec in the cathodal group and 25.24 o/sec in the anodal group pre-tDCS. Post-tDCS, the peak mean SPV for right cold irrigations was 16.25 o/sec in the cathodal group and 24.70 o/sec for the anodal group. For left cold irrigations post-tDCS, the mean peak SPV was 16.91 o/sec in the cathodal group and 25.53 o/sec for the anodal group. These recordings now allowed for the calculation of the nystagmus suppression index. We only observed a significant suppression of vestibular nystagmus following cathodal stimulation of the left posterior parietal cortex for both right (p=6.07x10-7, paired t-test) and left irrigations (p=5.35x10-7, paired t-test), as shown in Figure 1B. In contrast, anodal stimulation did not induce a significant vestibular nystagmus suppression for neither left (p=0.79, paired t-test) nor right (p=0.13, paired t-test) irrigations."

Q3. I am missing a hypothesis and predictions based on the hypothesis stated.
R3. In accordance with the reviewers request we now explicitly state our hypothesis and prediction in the introduction section of the manuscript which can be found on pages 3 and 4 of the revised manuscript. Alternatively, please see below.
"Previous findings have also separately revealed the functional importance of hemispheric lateralisation for cortical processing of both vestibular and anxiety signals(Balaban, 2002; Carmona et al., 2009). With respect to anxiety, the valence theory of emotional processing stipulates that the right hemisphere is specialized for the processing of negative emotions whereas the left hemisphere predominantly processes positive emotions (Ehrlichman, 1987; Silberman & Weingartner, 1986). Similarly, lateralisation of cortical processing has also been demonstrated in the vestibular system, and functionally we have recently illustrated the role of such right hemispheric vestibulo-cortical dominance upon the modulation of vestibular-guided behaviour, for both brainstem (Vestibulo-ocular reflex) and cortically mediated (vestibulo-perceptual) vestibular thresholds.
Based upon the above reviewed findings, we propose that hemispheric lateralisation acts as a possible link between anxiety networks and the vestibular system. Specifically, we postulate that an individual's level of non-situational (trait) anxiety may be linked to the extent of lateralisation of the vestibular cortex in terms of the degree of right hemisphere vestibulo-cortical dominance. In order to test

this prediction, we assessed both non-situational anxiety levels and the degree of right hemispheric vestibulo-cortical dominance in healthy individuals. Anxiety levels were determined using the trait component of the Spielberger State-Trait Anxiety inventory (Spielberger, C. D. et al 1980). Hemispheric lateralisation of the vestibular system was determined using a biomarker that assesses the degree to which a caloric-induced vestibular nystagmus is suppressed following modulation of cortical excitability using trans-cranial direct current stimulation (tDCS) applied over the posterior parietal cortex (Arshad et al., 2015), which is a key area in the vestibular cortical network. In conjunction, these measures will allow us to gage the nature of the relationship between anxiety networks and the vestibular system."

Q4. Page 4, third paragraph: "Irrigations were performed in both right and left ears with cold water (30oC) for 40s ..." This sentence might be misleading. I assume that both right and left ears refers to sequential caloric irrigation, not bilateral caloric irrigation at the same time.
R4. The reviewer is correct that the sentence highlighted may be misconstrued as to imply the caloric irrigations were applied bilaterally. However, this was not the case as the cold water irrigations were applied separately for the right and left ear respectively. We have now rephrased this sentence to clarify this point, and this amendment can be found on page 6, paragraph 2 of the revised manuscript. Alternatively, please see below for the implemented change.
"Irrigations were performed separately for both right and left ears, implementing a 5 minute rest period between irrigations to avoid any carry-over effects, with cold water (30oC) for 40s with a flow rate of 500ml/min. "
Q5. Page 5, 2nd paragraph: How did the authors evaluate psychiatric disorders in the controls before inclusion to the study?
R5. Prior to subjects participating in the experiment, we screened all the controls for any ophthalmological, neurological, psychiatric and otological disorders. Practically this was done by explicitly asking participants if they have ever seen or currently were seeing a medical specialist for any eye, ear, brain or psychiatric problem/ disorder. If the answer was yes to any of those questions, those subjects were automatically excluded from the study. We have clarified this point, on page 4, paragraph 2 of the revised manuscript as set out below.
"Subjects were excluded on a basis of any electric stimulation contraindications such as a personal or family history of epilepsy or any metal implants. Further, subjects were excluded if there was any past or existing psychiatric, otological, ophthalmological or neurological disorder. Practically this was performed during a screening session before participants were recruited into the study by asking them directly, if they were currently or ever had been in the past, under the care of a specialist doctor for any eye, ear, brain or psychiatric condition."
Q6. I am worried about the scores in the Trait Spielberger questionnaire reported. With more than 25% of the healthy controls having a score that indicates anxiety disorder, the questionnaire used is either not suitable or the cut-off point selected is too low. Also I wonder why the authors emphasize the value of 65. Is this another cut-off point? Also on, Page 5, 2nd paragraph: „Critically, there was no significant difference in Trait anxiety between the two groups (p>0.05, unpaired t-test)." is this referring to the 22 patients with anodal vs. the 22 patients with cathodal stimulation?
The cut of that is suggested in the literature is a score of 39/40 and it is the case that 25/64 subjects in our sample had a score >40. As aforementioned, the cut-off point selected was based on previous findings that have suggested the implementation of this value as a cut-off (Knight et al., 1983; Julian, 2011). However, the fact that individuals had high trait anxiety scores (i.e. >40) does not imply that that they have a clinically significant anxiety disorder but rather that they are more prone to anxious traits, as discussed on page 9 paragraph 1 of the revised manuscript. Alternatively, please see the inserted paragraph in red below. Regarding the second point of emphasizing the value of 65- this was simply included to state that this was the highest score obtained in the Trait Spielberger questionnaire in our sample, but admittedly it is confusing and we have now removed any reference to it in the revised manuscript.
"It is important to recall that in the current study we only tested normal subjects. Considering a suggested cut-off point of 39-40 in order to detect clinical manifestations of anxiety in the Trait Spielberger Questionnaire(Knight et al., 1983; Julian, 2011), of our 64 participants, 25 (N.B. 12 in the cathodal group and 13 in the anodal group), had a score higher than 40. The fact that these healthy controls have a score higher than the suggested cut-off, does not imply that there is an undetected anxiety disorder present, but simply that these subjects are more anxious on a day-to-day basis but are able to function normally. Critically, there was no significant difference in Trait anxiety scores when comparing between the 32 subjects in the experimental group (cathodal stimulation) and the 32 subjects in the control group (anodal stimulation) ((p= 0.276, paired t-test)."

Q7. If the authors do the correlation analysis again with the 10 subjects that had normal scores in the experimental group, is the reported correlation still present? This would help in confirming the correlation reported here in healthy control subjects.
R7. For the reviewer's and our own curiosity, we removed the highly anxious people from the original data set (previous submission without the additional subjects) and still observed a significant correlation ($R2=0.356$; $p< 0.01$).
Q8. How was the distribution of scores amongst these two groups?
R8: The table inserted below illustrates the distribution of the trait anxiety scores amongst the cathodal (experimental) and anodal (control) groups. Despite the mean score being higher in the anodal group, there was no significant difference between the overall trait anxiety scores when comparing both groups (p=0.277, paired t-test), as stated on page 9 of the revised manuscript.

Score Ranges	Frequency	
	Cathodal	Anodal
20-30	13	8
30-40	7	11
40-50	8	9
50-60	3	4

EJN European Journal of Neuroscience FENS Federation of European Neuroscience Societies

60-70 1 0
70-80 0 0
Mean Score 34.9 37.9

Q9. Page 6, 2nd paragraph from bottom: „...which should be further explored as a potential therapeutic option, especially in patients with chronic dizziness“ this conclusion comes out of the blue suddenly, as the authors speak about the treatment of chronic dizziness. Please clarify.
R9. We agree with the reviewer that this statement appears out of the blue and is somewhat distracting to the main message of our manuscript. Accordingly we have now completely removed the paragraph that discussed the use of tDCS as a potential treatment strategy for patients with chronic dizziness.
Q10. Figure legend Fig. 1: I am missing a description of the methods used to perform the regression analysis. Was an approach used that takes the fact into account that both variables were dependent, i.e. measured with noise? Normal regression analysis does require one independent variable.
R10: We performed a linear regression analysis with the Nystagmus Suppression Index as the independent variable on SPSS 24. This is reported as the R2 value and the corresponding significance throughout the manuscript. Further, we do not believe that both variables were dependent, as we propose that vestibulo-cortical hemispheric dominance of an individual impacts upon an individual's trait anxiety level, as opposed to trait anxiety determining vestibulo-cortical dominance.

Reviewer: 2; This study investigates the relationship between hemispheric dominance in vestibular processing and anxiety trait. Vestibular-caloric nystagmus suppression induced by left anodal transcranial direct current stimulation positively correlated with anxiety questionnaire scores. The results seem to suggest that stronger right-hemispheric specialization relates to lower trait anxiety. The research question is interesting and novel. However, the manuscript presents with deficiencies in experimental design and over-interpretation of the results. I do not recommend publication of the manuscript, and dataset, in its current version.
We thank the reviewer for his/her overall positive assessment of our manuscript. Regarding the limitations identified, we have now addressed the concerns raised by the reviewer on a point by point basis, as set out below.
Q1. A major concern is that vestibular stimulation (and/or TDCS) may have affected questionnaire ratings. The authors presented questionnaires either before or after vestibular/TDC stimulation to subjects who received either anodal or cathodal TDCS. Results were analyzed across subjects separately for the anodal and cathodal group. Was there a difference in questionnaire scores between patients receiving the questionnaire before or after vestibular stimulation? Also between those receiving anodal vs. cathodal stimulation when completing the questionnaire after the experiment? Further, was the proportion of subjects who received the questionnaire before vs. after balanced in the anodal vs. cathodal groups? These possible confounds could have been addressed by administering the questionnaire twice (before and after) the experiment. Post-hoc analysis, giving matched and sufficiently large sample sizes across subgroups should be included.
R1. In both groups (i.e. anodal and cathodal) half the subjects completed the questionnaire before the application of any tDCS and vestibular stimulation and the remaining half of the subjects completed the questionnaire after the application of tDCS and vestibular stimulation (i.e. the proportion of subjects that received the questionnaire before and after tDCS &vestibular stimulation was balanced). Further, to specifically address the concern raised by the reviewer we have adopted his/her suggestion to apply a post hoc analysis. An unpaired t-test comparing questionnaire scores for anxiety before and after tDCS and vestibular stimulation revealed no difference (P> 0.05 t-test). We clarify this issue and report this result point on page 5, paragraph 2 (middle) of the revised manuscript. Alternatively, please see below for the implemented change.
"Since the Trait component assesses stable rather than situational anxiety levels, the experimental protocol implemented (see below) should not impact anxiety scores. However, in order to explicitly eliminate any potential carry-over effects of the experimental protocol (i.e. either brain stimulation or vestibular stimulation), half the participants in both the anodal and cathodal groups completed the questionnaire before and the other half after the experiment. Note, we observed no significant difference between these two groups when comparing the scores obtained either before or after vestibular and brain stimulation respectively (p>0.05 , unpaired t-test)."

Q2. Another concern is that authors exclusively applied TDCS to the left hemisphere; however, results are discussed with respect to (not tested) right hemispheric processing. It may be one of many possible causes underlying the observed effect, however, based on unilateral left stimulation alone this conclusion cannot be drawn. A cross-over design including unilateral left, unilateral right, as well as bilateral stimulation (as used in a previous publication by the same authors, Arshad et al. 2014 Brain Stimulation). Also, the authors should expand the introduction of the background for the left TDCS technique testing hemispheric dominance (e.g. see Arshad et al. 2014 Brain Stimulation).
R2. The reviewer is correct in that we only applied tDCS over the left hemisphere. It is important to recall that in right-handed individuals there exists right hemisphere dominance for vestibular cortical processing (Dieterich et al., 2003, Arshad et al; 2013, Arshad et al., 2017. Lopez et al., 2012, Zu Eulenburg et al., 2012). Specifically, we have previously shown that cathodal stimulation of the left parietal cortex resulted in bilateral but asymmetrical reduction in vestibular nystagmus (i.e. greater reduction in the peak slow phase velocity of left-beating nystagmus compared to changes in velocity for right-beating nystagmus). These previous findings, suggest that left cathodal stimulation inhibits the left hemisphere, potentiating right hemisphere dominance (N.B. to a greater extent in more right hemisphere dominant individuals), and thus rendering the left hemisphere less able to process the vestibular nystagmus) Arshad et al., 2014). Accordingly, greater vestibular nystagmus suppression following left parietal cathodal tDCS implies increased right hemispheric vestibular dominance, albeit somewhat paradoxically that the stimulation was applied over the left hemisphere (Arshad et al., 2015).

Further, as pointed out by the reviewer, in our previous work we have shown that changing excitability (either through facilitation via anodal stimulation or inhibition via cathodal stimulation) of the right hemisphere does not have any subsequent modulatory effect upon the VOR (Arshad et al., 2014). We propose that such absence of modulation following right hemisphere stimulation is attributable to the fact that in right handers, the right hemisphere is able to better compensate than the left for the induced inhibition via cathodal stimulation, thanks to its superior preponderance for vestibular processing (Dieterich et al., 2003; Arshad et al., 2014). An alternative, but not a mutually exclusive explanation, is that there is an on-going functional asymmetry between the two parietal cortices; hence, the right hemisphere exerts stronger inhibition over the left hemisphere, a notion supported by the previous findings demonstrating that parietal interhemispheric connections are asymmetric (Koch et al., 2011). We address the above points in a summarised version on pages 6 and 7 of the revised manuscript, as set out below.
"To explain the choice of the implemented stimulation paradigm further, it is beneficial to briefly review our previous findings in a sequential order. Application of a bi-hemispheric montage, namely anodal stimulation of the right hemisphere (posterior parietal cortex; P3/P4) and concurrent cathodal stimulation of the left hemisphere results in suppression of left-beating vestibular-nystagmus (right-ear cold irrigations). This nystagmus is predominantly processed by the left hemisphere, but this bi-hemispheric montage has no effect upon the right beating nystagmus produced by left-ear cold water irrigations, which are predominantly processed in the right hemisphere. Reversing the polarities in the bi-hemispheric montage has no modulatory effect upon the VOR (Arshad et al., 2014). To elucidate the activate electrode in the bi-hemispheric montage, we applied unipolar tDCS and observed that the modulation was attributable to cathodal stimulation of the left hemisphere.

Critically, our previous results demonstrate that right hemisphere cathodal stimulation does not modulate the vestibulo-ocular reflex in right handed individuals, and this lack of modulation is surprising since inhibition of the right posterior parietal cortex disrupts inter-hemispheric interactions and should theoretically affect vestibular processing(Arshad et al., 2014). However, this lack of modulation can be reconciled with the notions that in right handers, the right hemisphere is able to better compensate than the left for the induced inhibition via cathodal stimulation, attributable to its dominance for vestibular processing (Dieterich et al., 2003; Arshad et al., 2014), and the on-going functional asymmetry between the two parietal cortices enabling the right hemisphere to exert stronger inhibition over the left hemisphere facilitated by asymmetric parietal interhemispheric connections (Koch et al., 2011).

Accordingly, our previous findings imply that cathodal stimulation over the left hemisphere, not only inhibits function in the left hemisphere but also potentiates right hemisphere dominance, and thus renders the left hemisphere less able to process the vestibular nystagmus. Thus, greater vestibular nystagmus suppression following left parietal cathodal tDCS implies increased right hemispheric vestibulo-cortical dominance."

Q3. page 5: "Our results demonstrate that … hemispheric dominance can predict anxiety levels". No, "prediction" implies temporal precedence, which cannot be inferred from the correlational results presented here.
R3. We accept the reviewer's point that, "predict" is the wrong choice of terminology. Accordingly, we have now changed this in the revised manuscript so that it reads, "hemispheric dominance was found to correlate with an individual's anxiety levels".
Q4. page 6: ".. we observed that a more right hemisphere dominant individual is better able to control negative emotions..". It is not clear if the questionnaire event tested the control of negative emotions. Authors should report the questionnaire questions, and analysis of sub-scores in order to support their claim.
R4. We agree with the reviewer that using the phrase, "better able to control negative emotions", is rather ambiguous and according we have now dropped this and replaced with what we actually found. That is, our results suggest that greater right-hemispheric vestibular specialization relates to lower trait anxiety". Further, as requested we now detail more carefully aspects of the questionnaire questions implemented on page 5, paragraph 2 of the revised manuscript. Alternatively, please see below for the changes.
"The Trait component questionnaire contains 20 statements ascertaining various aspects of an individual's propensity to anxious traits(Julian, 2011). Subjects were asked to rate the 20 questions on a scale of 1-4 (with 1=never, 2=sometimes, 3=very often, 4=always). Questions are aimed at assessing feelings of stress, worry and other manifestations of anxiety on a day to day basis, for example, 'I feel like a failure', 'I am happy' or 'I worry too much about things that really doesn't matter'(Spielberger & Sydeman, 1994). Accordingly, the questionnaire yields a maximum score of 80 and a minimum of 20, with higher scores representing greater levels of anxiety."

Q5. Our findings suggest that hemispheric dominance is the intricate mechanistic link between anxiety networks and the vestibular system. ". Based on a rather vague discussion of hemispheric dominance, and the aforementioned concerns, it is unclear how the authors arrive at this conclusion - mechanistic, how exactly? Also it, would have been desirable in the introduction to provide evidence to the hemispheric specialisation hypothesis to be tested. That is, the Introduction is rather weak, as it remains vague at pointing out the involvement of cortical hemispheres, without clearly showing that the same hemisphere and the same functional networks are involved.
R5. Firstly, we apologise for the over interpretation of our findings. We now state that, "our findings imply the right hemispheric vestibular specialization relates to lower trait anxiety". Further, in accordance with the reviewers request we now explicitly state our hypothesis and prediction in the introduction section of the manuscript regarding how hemispheric dominance mechanistically links the anxiety networks and the vestibular system, which can be found on pages 3 and 4 of the revised manuscript. Alternatively, please see below.
"Previous findings have also separately revealed the functional importance of hemispheric lateralisation for cortical processing of both vestibular and anxiety signals(Balaban, 2002; Carmona et al., 2009). With respect to anxiety, the valence theory of emotional

EJN European Journal of Neuroscience FENS Federation of European Neuroscience Societies

processing stipulates that the right hemisphere is specialized for the processing of negative emotions whereas the left hemisphere predominantly processes positive emotions (Ehrlichman, 1987; Silberman & Weingartner, 1986). Similarly, lateralisation of cortical processing has also been demonstrated in the vestibular system, and functionally we have recently illustrated the role of such right hemispheric vestibulo-cortical dominance upon the modulation of vestibular-guided behaviour, for both brainstem (Vestibulo-ocular reflex) and cortically mediated (vestibulo-perceptual) vestibular thresholds.
Based upon the above reviewed findings, we propose that hemispheric lateralisation acts as a possible link between anxiety networks and the vestibular system. Specifically, we postulate that an individual's level of non-situational (trait) anxiety may be linked to the extent of lateralisation of the vestibular cortex in terms of the degree of right hemisphere vestibulo-cortical dominance. In order to test this prediction, we assessed both non-situational anxiety levels and the degree of right hemispheric vestibulo-cortical dominance in healthy individuals. Anxiety levels were determined using the trait component of the Spielberger State-Trait Anxiety inventory (Spielberger, C. D. et al 1980). Hemispheric lateralisation of the vestibular system was determined using a biomarker that assesses the degree to which a caloric-induced vestibular nystagmus is suppressed following modulation of cortical excitability using trans-cranial direct current stimulation (tDCS) applied over the posterior parietal cortex(Arshad et al., 2015), which is a key area in the vestibular cortical network. In conjunction, these measures will allow us to gage the nature of the relationship between anxiety networks and the vestibular system."

Q6. "left posterior parietal cortex, an area heavily implicated in the processing of vestibular signals" is somehow at odds with the notion of right-hemispheric dominance (Dieterich et al. 2003 Cerebral Cortex)
R6. We acknowledge the reviewer's point that the inserted word, "left", in the context of the highlighted sentence is confusing and accordingly we have now omitted it.
Q7. page 2: "… hemispheric lateralization determines … emotional processing.": The results are correlational and cannot be interpreted in terms of causality.
R7. We agree with the reviewer's concern and have changed this as set out below or alternatively please refer to pages 9 and 10 of the revised manuscript.
"The role of hemispheric lateralisation in emotional processing has been repeatedly demonstrated in the literature. Lesion studies have shown that left hemisphere damage is associated primarily with depressive symptoms(Bolla-Wilson et al., 1989; Starkstein et al., 1989). Contrastingly, individuals with right hemisphere lesions reported difficulties in emotional facial recognition tasks(Mandal et al., 1996; Bourne & Watling, 2015). Further, the right hemisphere has been implicated both in the major processing and displaying of all affective states as demonstrated by more intense facial expression of emotion on the left side of the face (Sackeim et al., 1978; Killgore & Yurgelun-Todd, 2007; Alves et al., 2008). More specifically, patients with brain tumours in the right hemisphere score more highly on anxiety scales than patients with left hemisphere tumours. Notably, following tumour removal the elevated anxiety scores return to baseline values (Mainio et al., 2003). These findings are supported by the valence theory of emotional processing, which proposes the predominant processing of negative emotions, such as fear, in the right hemisphere, whilst positive emotions, such as happiness, are processed in the left hemisphere(Davidson, 1995). Thus, it appears to be the case that disruption of normal hemispheric interactions results in maladaptive emotional processing functionally manifesting as anxiety (Davidson et al., 1992)."
Q8. page 3. "The link between anxiety and vestibular system, …". It appears the authors refer to two distinct types of systems? Vestibular system, a sensory system processing vestibular afferents, and the anxiety system attributing negative valence to a large variety of sensory inputs?
R8. The reviewer is absolutely correct that we are referring to two distinct systems and have now clarified our use of language to ensure that it is absolutely clear that these two systems, namely the vestibular system and the anxiety system are distinct. This change is implemented throughout the entire revised manuscript.
Q9. page 4. Did the authors not administer the "State" component of the questionnaire or missed to report these results? Could thxey include a listing of all questions, median scores, interquartile range across subjects, and report analysis of sub-scores (e.g. emotion control).
R9. Unfortunately, in the present study we did not administer the "state" component of the questionnaire, as we were specifically interested in the stable rather than situational anxiety levels of an individual.
Q10. Authors should also analyse and report peak latency of the slow phase velocity within the 2min recording period between pre- and post-TDCS. Further, how was the nystagmus suppression index exactly calculated? by dividing the differences post minus pre by the absolute value of pre, multiplied by 100? Could the authors specify this in the manuscript?
R10. We apologise for this omission, and now report this in the last paragraph of page 7 and the first paragraph of page 8 of the manuscript, or alternatively, please refer to the inserted paragraph below. Further, we have summarised this data graphically in Figure 1B and we now also show illustrations using raw traces of the vestibular nystagmus suppression following cathodal stimulation (Figure 1A).
"Firstly, it is important to confirm a significant impact of tDCS upon vestibular function, specifically assessed by the suppression of the mean peak SPV of a caloric-induced nystagmus, as illustrated by the raw vestibular nystagmus traces from one of our participant's (Figure 1A). Pre-tDCS, the mean peak SPV for the right cold irrigations was 28.28 o/sec in the cathodal group and, 25.03 o/sec in the anodal group. For left cold irrigations the mean peak SPV was 25.23 o/sec in the cathodal group and 25.24 o/sec in the anodal group pre-tDCS. Post-tDCS, the peak mean SPV for right cold irrigations was 16.25 o/sec in the cathodal group and 24.70 o/sec for the anodal group. For left cold irrigations post-tDCS, the mean peak SPV was 16.91 o/sec in the cathodal group and 25.53 o/sec for the anodal group. These recordings now allowed for the calculation of the nystagmus suppression index. We only observed a significant suppression of vestibular nystagmus following cathodal stimulation of the left posterior parietal cortex for both right (p=6.07x10-7, paired t-test) and left irrigations (p=5.35x10-7, paired t-test), as shown in Figure 1B. In contrast, anodal stimulation did not induce a significant vestibular nystagmus suppression for neither left (p=0.79, paired t-test) nor right (p=0.13, paired t-test) irrigations."

Q11; page 5. Could the authors perform additional correlation analysis between the SP peak velocity before TDCS and questionnaire scores, and a separate analysis of SP peak velocity after TDCS and questionnaire scores. No correlation in these analyses would further strengthen support the interpretation that TDCS-induced suppression relates to anxiety scores. However significant correlation with pre-TDCS SPV values could reflect TDCS unrelated effects that may have contributed to the pre-post change index (if used to normalize this difference value).
R11. In accord with the reviewer's request, we have now performed this additional analysis. We observed no relationship between the slow phase velocities before tDCS and the questionnaire scores in neither the cathodal nor the anodal group. However, we did observe a significant correlation between post tDCS SPV in the cathodal group but not the anodal group. We now report the results of this additional analysis on Page 8, paragraph 2 of the revised manuscript. Alternatively, please refer below for the implemented change. .
"We observed a significant positive correlation was observed (Pearson's correlation coefficient= R2 0.525, p= 0.000003; Figure 1C) in the experimental group (i.e. cathodal stimulation). That is, more right hemisphere dominant participants (i.e. greater nystagmus suppression) scored lower on the Trait Anxiety Questionnaire. Less right hemisphere dominant individuals (i.e. lower nystagmus suppression) scored more highly on the questionnaire. As expected, no correlation was observed in the control group [i.e. anodal stimulation] (Pearson's correlation coefficient= R2 0.005, p=0.687; Figure 1D). Further, supporting the relationship we report is the fact that we observed no significant correlation between trait anxiety scores and pre-cathodal tDCS peak mean SPV for either right cold (Figure 1E, R2= 0.01939 p=0.447) or left cold irrigations (Figure 1E, R^2 = 0.09075 p=0.094). Post-tDCS a significant correlation can be seen between trait anxiety scores and the peak mean SPV for both right cold (Figure 1F, R2=0.26436, p= 0.002610) and left cold irrigations (Figure 1F, R2=0.38605, p= 0.000148). One would expect a weak correlation to exist between trait anxiety and the mean peak SPV following the application of tDCS, as this reflects the cortical influences of tDCS. This correlation is strengthened by the full calculation of the nystagmus suppression index by incorporating the pre-tDCS SPV values."

Q12. Figure 1. It seems preferable to show the y-axis on the left-hand side of the plot. Also the same plot should be added for the other (control) group.
In accordance with the reviewers request we now represent the results for anodal group as well as the cathodal group in Figure 1C and 1D. Further we have also added the y-axis on the left hand-side as requested.

2nd Editorial Decision 26 February 2018

Dear Dr. Arshad,

Your resubmitted paper has been evaluated by one of the original reviewers and by a new reviewer who had access to the previous reviews. As you will see, this new reviewer expresses concerns about the mechanistic explanation based on correlation analysis and requests more careful interpretation and discussion of the results. If you can be responsive to this last remaining issue, we will be pleased to accept the paper for publication here at EJN at that stage.

When revising your manuscript, please also attend to the following editorial issues:

1. Please ensure that you provide a Graphical Abstract (see instructions at the end of this email for guidance on how to prepare this).

2. Please ensure that your manuscript contains the mandatory Data Accessibility statement.

3. Please note that sub-figures must not have individual captions. Just one caption should be provided for the figure as a whole.

4. For improved readability, we suggest that you consider breaking the figure down into a few separate figures.

Thank you for submitting your work to EJN.

Kind regards,

John Foxe & Paul Bolam
co-Editors in Chief, EJN

Reviews:
Reviewer: 1 Alexander Tarnutzer (University Hospital Zurich, Switzerland)
Comments to the Author
All my comments and concerns have been appropriately addressed.

Reviewer: 3 Fred Mast (University of Lausanne, Switzerland)

Comments to the Author
This is a revision of a paper that received previous reviews already, and I joined in as yet another reviewer. Previous reviews and manuscripts were made available. Even though this submission is a short paper, the meanwhile accumulated reading materials were quite extensive, and I apologize for the delay. I think the manuscript improved and much of what Reviewer 2 suggested was implemented in the revised version. In particular, the authors now widely refrain from overstating their findings. They also included more participants, and this also resolved some of the concerns. Still, however, the authors use the term "mechanistic". But what is actually the mechanism that they are showing here? This requires some more to-the-point elaboration even though space is restricted for a short paper. It is a correlational study, and this is why any "mechanistic" explanation is hard to support. Indeed, what I find puzzling about this manuscript is the absence of any explanation about how VOR modification can be used as an index for the vestibulo-affective connections. In other words, excitability changes of the parietal network are likely to alter a cortical measure of vestibular perception. And in this sense, I would have rather expected a correlation between between a cortical measure and the anxiety questionnaire. The VOR changes are more remote from the explicit nature of the questionnaire and therefore it requires more explanation about how the excitability changes induced by tDCS changes the vestibular cortical network and how this in turn alters VOR characteristics. VOR and perceptual measures often dissociate, and therefore, it is not clear why the VOR should be a good indicator of affective measures. If, however, there is empirical evidence providing support it needs to be cited. There is no doubt: The fact that left parietal tDCS has altered VOR characteristics is by itself an interesting finding. However, the authors need to add an explantion about the potential "mechanism" rather than claiming the existence thereof. This will in turn help to better understand how the processing of vestibular information can relate to the anxiety questionnaire. Referring to the literature is not enough. This is, in brief, what I was missing in this paper. I found the approach by all means novel because it combines caloric stimulation with tDCS.

Authors’ Response 09 March 2018

Reviewer: 3 Comments to the Author

This is a revision of a paper that received previous reviews already, and I joined in as yet another reviewer. Previous reviews and manuscripts were made available. Even though this submission is a short paper, the meanwhile accumulated reading materials were quite extensive, and I apologize for the delay. I think the manuscript improved and much of what Reviewer 2 suggested was implemented in the revised version. The authors now widely refrain from overstating their findings. They also included more participants, and this also resolved some of the concerns. I found the approach by all means novel because it combines caloric stimulation with tDCS.
We thank the reviewer for taking the time to review our manuscript and his/her positive and insightful comments. Please find below a point by point response to the criticisms raised below.
Q1. Still, however, the authors use the term "mechanistic". But what is actually the mechanism that they are showing here? It is a correlational study, and this is why any "mechanistic" explanation is hard to support.
R1. We apologise for this. In agreement with the reviewer we have now removed any reference to mechanistic. Please refer to the abstract and discussion sections of the manuscript for the implemented changes, highlighted in red.
Q2. Indeed, what I find puzzling about this manuscript is the absence of any explanation about how VOR modification can be used as an index for the vestibulo-affective connections. In other words, excitability changes of the parietal network are likely to alter a cortical measure of vestibular perception. And in this sense, I would have rather expected a correlation between a cortical measure and the anxiety questionnaire. The VOR changes are more remote from the explicit nature of the questionnaire and therefore it requires more explanation about how the excitability changes induced by tDCS changes the vestibular cortical network and how this in turn alters VOR characteristics. VOR and perceptual measures often dissociate, and therefore, it is not clear why the VOR should be a good indicator of affective measures. If, however, there is empirical evidence providing support it needs to be cited. There is no doubt: The fact that left parietal tDCS has altered VOR characteristics is by itself an interesting finding. However, the authors need to add an explanation about the potential "mechanism" rather than claiming the existence thereof. This will in turn help to better understand how the processing of vestibular information can relate to the anxiety questionnaire. Referring to the literature is not enough. This is, in brief, what I was missing in this paper.
R2. The reviewer is correct that vestibular ocular and perceptual measures often dissociate and that it is somewhat paradoxical that the brainstem mediated vestibular-ocular reflex should provide a good indicator of affective measures. However, it is important to note as shown in Figure 1E, no relationship exists between baseline measures of VOR and the affective measure. Rather, it is the degree of nystagmus suppression following left cathodal tDCS (i.e. top-down cortical modulation of the VOR) that correlates with the affective measure. We have previously shown that this cortically mediated modulation of the brainstem VOR correlates with cortical biases in numerical cognition (Arshad et al., 2016 Cerebral Cortex) and spatial attentional biases (Arshad et al., 2015 Neuroscience). Thus, the nystagmus suppression index reflects a cortical measure that is exerted on the brainstem mediated VOR. We suggest that this allows brainstem and cortical estimates of self-displacement to be synchronised and reflects a Brainstem-Cortical Scaling Metric (BCSM). Accordingly, we have added the paragraph inserted below to clarify this point in the discussion section on pages 10 and 11 of the revised manuscript.

EJN European Journal of Neuroscience FENS Federation of European Neuroscience Societies

“An outstanding question is why an association exists between a low-level brainstem-mediated VOR and anxiety, an affective measure. To address this, it is firstly important to note that the baseline measures of the VOR do not correlate with anxiety scores, as shown in Figure 1E. Rather, it is the degree of cortically mediated suppression of the brainstem vestibular-ocular reflex that correlates with the anxiety scores, implying a cortically-controlled re-scaling of brainstem-cortical interactions. In line with this, our previous findings have demonstrated that the nystagmus suppression index correlates with cognitive biases such as numerical magnitude perception and spatial attention(Arshad et al., 2015, 2016), further supporting the notion of a brainstem-cortical re-scaling network.”
